# The threonine degradation pathway of the Trypanosoma brucei procyclic form: the main carbon source for lipid biosynthesis is under metabolic control

**DOI:** 10.1111/mmi.12351

**Published:** 2013-08-25

**Authors:** Yoann Millerioux, Charles Ebikeme, Marc Biran, Pauline Morand, Guillaume Bouyssou, Isabel M Vincent, Muriel Mazet, Loïc Riviere, Jean-Michel Franconi, Richard J S Burchmore, Patrick Moreau, Michael P Barrett, Frédéric Bringaud

**Affiliations:** 1 Centre de Résonance Magnétique des Systèmes Biologiques (RMSB), UMR-5536 Université Bordeaux Segalen, CNRS146 rue Léo Saignat, 33076, Bordeaux, France; 2 Laboratoire de Biogenèse Membranaire, UMR-5200 Université Bordeaux Segalen, CNRSBâtiment A3-1er étage, INRA Bordeaux Aquitaine BP81, 71 avenue Edouard Bourlaux, 33883, Villenave d'Ornon cedex, France; 3 Wellcome Trust Centre for Molecular Parasitology, and Glasgow Polyomics, Institute of Infection, Immunity and Inflammation, CMVLS, University of GlasgowGlasgow, G12 8TA, UK; 4 Laboratoire de Microbiology Cellulaire et Moléculaire et Pathogénicité, UMR-5234 Université Bordeaux Segalen, CNRS146 rue Léo Saignat, 33076, Bordeaux, France

## Abstract

The *Trypanosoma brucei* procyclic form resides within the digestive tract of its insect vector, where it exploits amino acids as carbon sources. Threonine is the amino acid most rapidly consumed by this parasite, however its role is poorly understood. Here, we show that the procyclic trypanosomes grown in rich medium only use glucose and threonine for lipid biosynthesis, with threonine's contribution being ∼ 2.5 times higher than that of glucose. A combination of reverse genetics and NMR analysis of excreted end-products from threonine and glucose metabolism, shows that acetate, which feeds lipid biosynthesis, is also produced primarily from threonine. Interestingly, the first enzymatic step of the threonine degradation pathway, threonine dehydrogenase (TDH, EC 1.1.1.103), is under metabolic control and plays a key role in the rate of catabolism. Indeed, a trypanosome mutant deleted for the phosphoenolpyruvate decarboxylase gene (*PEPCK*, EC 4.1.1.49) shows a 1.7-fold and twofold decrease of TDH protein level and activity, respectively, associated with a 1.8-fold reduction in threonine-derived acetate production. We conclude that TDH expression is under control and can be downregulated in response to metabolic perturbations, such as in the PEPCK mutant in which the glycolytic metabolic flux was redirected towards acetate production.

## Introduction

Trypanosomes of the *Trypanosoma brucei* group are the aetiological agents of Human African trypanosomiasis, a parasitic disease that affects over 36 countries in sub-Saharan Africa (Barrett *et al*., 2003). The *T. brucei* life cycle is complex, and the parasite adapts to life in both its insect (tsetse fly) and mammalian hosts. These distinct environments require remodelling of parasite metabolism to enable adaptation in each. In the glucose-rich environment of mammalian blood, the bloodstream forms of the parasite rely solely on glucose to produce energy. The procyclic forms of the parasite living in the tsetse fly midgut – where glucose availability is scarce or absent – have developed an elaborate energy metabolism based on amino acids such as proline and threonine. In the insect, central energy metabolism of the procyclic trypanosomes is based on breakdown of proline, the main carbon and energy source circulating in haemolymph of the tsetse fly (Coustou *et al*., 2008). Threonine, however, is the most abundantly consumed carbon source in procyclics grown in glucose-depleted conditions (Lamour *et al*., 2005), but its contribution and exact role in central metabolism remains poorly understood.

**Figure 1:**
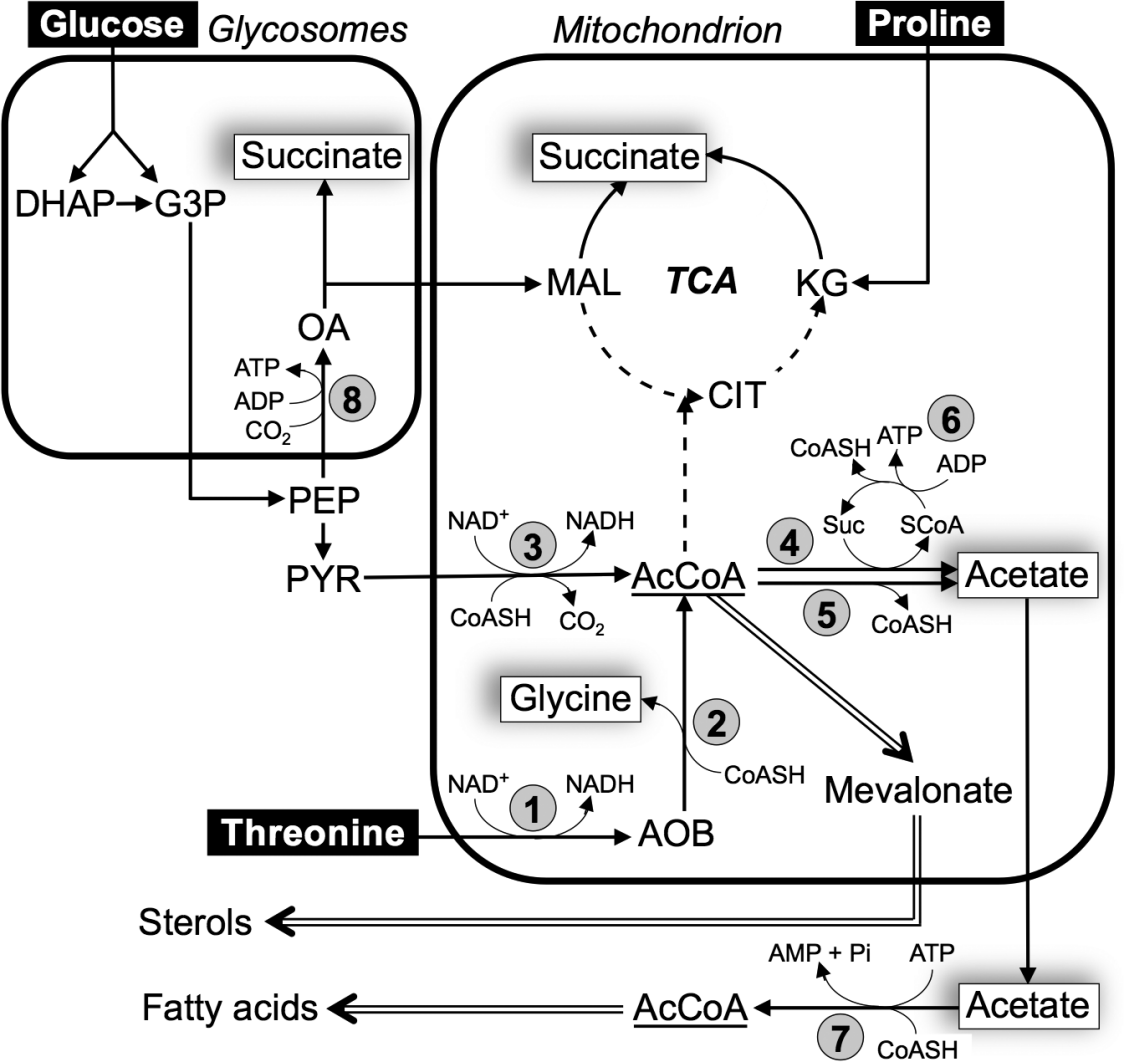
Schematic representation of acetate metabolism in procyclic trypanosomes grown in glucose-rich conditions. Black arrows indicate enzymatic steps of glucose, proline and threonine metabolism, dashed arrows symbolize steps supposed to occur at a background level or not at all, and double line arrows represent *de novo* sterol and fatty acid biosynthesis from acetyl-CoA. Excreted end-products of metabolism of glucose, proline and threonine are boxed. Indicated enzymes are: 1, threonine 3-dehydrogenase (TDH); 2, 2-amino-3-ketobutyrate coenzyme A ligase; 3, pyruvate dehydrogenase complex (PDH); 4, acetate:succinate CoA-transferase (ASCT); 5, acetyl-CoA thioesterase (ACH); 6, succinyl-CoA synthetase; 7, AMP-dependent acetyl-CoA synthetase (AceCS); 8, phosphoenolpyruvate carboxykinase (PEPCK). AcCoA, acetyl-CoA; AOB, amino oxobutyrate; CIT, citrate; DHAP, dihydroxyacetone phosphate; G3P, glyceraldehyde 3-phosphate; KG, 2-ketoglutarate; MAL, malate; OA, oxaloacetate; PEP, phosphoenolpyruvate; PYR, pyruvate.

When grown in standard glucose-rich conditions, procyclic trypanosomes prefer d-glucose to l-proline as a carbon source (Bringaud *et al*., 2006). As a consequence, the rate of proline consumption is up to sixfold reduced, with succinate becoming the main end-product of its metabolism, instead of alanine (Lamour *et al*., 2005; Coustou *et al*., 2008). Glucose becomes the major carbon source when present, being converted by aerobic fermentation to the partially oxidized end-products, succinate and acetate (Bringaud *et al*., 2012). The first seven steps of glycolysis are sequestered within a peroxisome-like organelle, called the glycosome (Opperdoes and Borst, 2012; Gualdron-Lopez *et al*., 2012). In the course of glycolysis, phosphoenolpyruvate (PEP) is produced in the cytosol, where it is located at a branching point to feed the glycosomal ‘succinate branch’ and the mitochondrial ‘acetate and succinate branches’ (Fig. 1). To produce succinate from glucose, PEP must re-enter the glycosomes where it is converted first to oxaloacetate by PEP carboxykinase (PEPCK, EC 4.1.1.49, step 8 in Fig. 1). PEP can also be converted to pyruvate, which enters the mitochondrion to feed the pyruvate dehydrogenase complex (PDH, EC 1.2.4.1, step 3) for acetyl-CoA production. Most, if not all, of the acetyl-CoA is then converted into excreted acetate by mitochondrial acetate:succinate CoA-transferase (ASCT, EC 2.8.3.8, step 4) and acetyl-CoA thioesterase (ACH, EC 3.1.2.1, step 5) (Van Hellemond *et al*., 1998; Riviere *et al*., 2004; Millerioux *et al*., 2012), a canonical tricarboxylic acid cycle being not operative in these cells (Van Weelden *et al*., 2003). ASCT contributes to an important part of mitochondrial ATP production through the ASCT/succinyl-CoA synthetase (EC 6.2.1.4) cycle (steps 4 and 6), while ACH does not (Bringaud *et al*., 2010; Tielens *et al*., 2010; Millerioux *et al*., 2012). In addition to ATP generation, mitochondrially derived acetate is essential to feed *de novo* fatty acid biosynthesis, through the trypanosomatid-specific acetate shuttle (Riviere *et al*., 2009). In this newly discovered pathway, acetate produced in the mitochondrion is converted back to acetyl-CoA in the cytosol by acetyl-CoA synthetase (AceCS, EC 6.2.1.1, step 7), to feed the initial cytosolic step of fatty acid biosynthesis (Riviere *et al*., 2009; Vigueira and Paul, 2011).

Threonine metabolism contributes to the production of acetate in trypanosomes (Cross *et al*., 1975; Linstead *et al*., 1977). According to the current model, this amino acid is converted to equal amounts of glycine and acetate in the mitochondrion, although the metabolic pathway leading to these excreted end-products has not been investigated using the tools of reverse genetics and metabolic profiling developed during the last decade. Here we have investigated the threonine degradation pathway in the procyclic trypanosomes, as well as the contribution of threonine to acetate and lipid production, using a combination of reverse genetics approaches [knockout and/or RNA interference (RNAi) downregulation], analyses of excreted end-products from these carbon sources by nuclear magnetic resonance (NMR) spectrometry and incorporation of radiolabelled carbon sources into lipids by high performance thin layer chromatography (HPTLC).

## Results

### Threonine is the main source of acetate production

**Figure 2:**
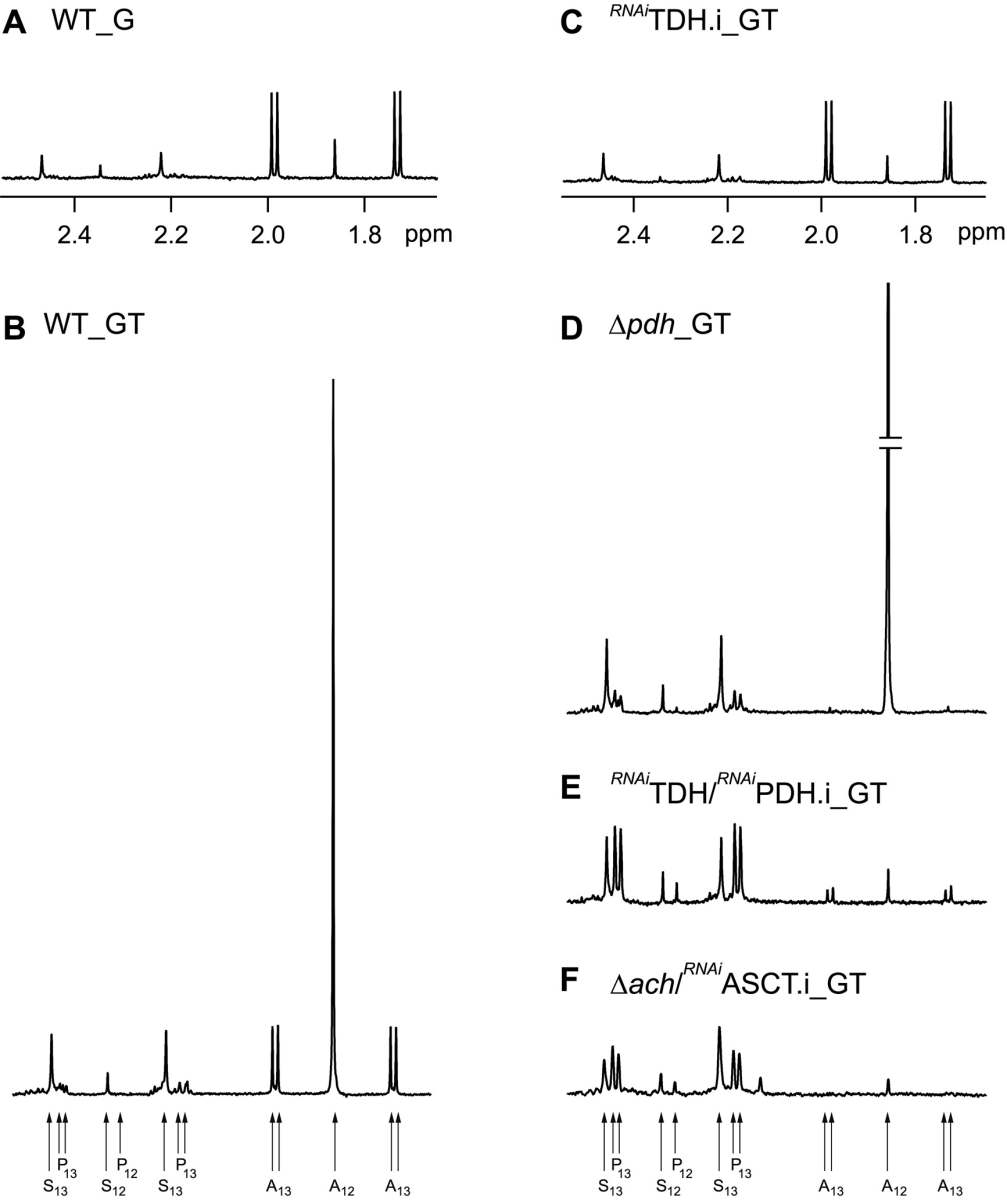
^1^H-NMR analysis of excreted end-products from glucose and threonine metabolism. Metabolic end-products (succinate, pyruvate, and acetate) excreted by the procyclic wild-type cell line (A and B), the Δ*pdh* mutant (D) and the tetracycline-induced ^*RNAi*^TDH.i (C), ^*RNAi*^TDH/^*RNAi*^PDH.i (E) and Δ*ach*/^*RNAi*^ASCT.i (F) mutants from d-[U-^13^C]-glucose and/or threonine was determined by ^1^H-NMR. The cells were incubated in PBS containing 4 mM d-[U-^13^C]-glucose with (_GT) or without (_G) 4 mM threonine. Each spectrum corresponds to one representative experiment from a set of at least 3. A part of each spectrum ranging from 1.6 ppm to 2.6 ppm is shown (see panels A and C). The resonances were assigned as indicated in panels B and F: A_12_, acetate; A_13_, ^13^C-enriched acetate; P_12_, pyruvate; P_13_, ^13^C-enriched pyruvate; S_12_, succinate; S_13_, ^13^C-enriched succinate.

It is currently accepted that the procyclic trypanosomes produce acetate from glucose and threonine (Bringaud *et al*., 2006); however, their relative contribution is unknown and possible involvement of additional carbon source(s) to acetate production has not been investigated. To address these questions we have developed a metabolite profiling assay based on the ability of proton NMR spectrometry to distinguish ^13^C-enriched from ^12^C molecules. Cells were incubated in PBS with equal amounts (4 mM) of d-[U-^13^C]-glucose and unenriched threonine (natural ^13^C-enrichment is 1.1%), in order to perform a quantitative analysis of threonine-derived and glucose-derived acetate production by ^1^H-NMR. In addition, the same experiment was performed without threonine, to compare d-[U-^13^C]-glucose metabolism in the presence and in the absence of the amino acid. When glucose is the only carbon source in the incubation medium, the procyclic trypanosomes excreted mainly acetate and succinate with traces of pyruvate from glucose metabolism (Fig. 2A and Table 1). [^13^C]-Acetate derived from d-[U-^13^C]-glucose (annotated A_13_ in Fig. 2B) is represented by two doublets, with chemical shifts at around 2.0 ppm and 1.75 ppm respectively (see Fig. 2A). The central resonance (1.88 ppm) corresponding to [^12^C]-acetate, probably derived from an unknown internal carbon source (11.4% of the excreted acetate; 193 nmol *versus* 1692 nmol of excreted molecules h^−1^ mg^−1^ of protein), is not included in the quantitative analyses. Addition of threonine to the d-[U-^13^C]-glucose/PBS medium induces (i) a huge increase of [^12^C]-acetate excretion (3348 nmol *versus* 193 nmol of excreted molecules h^−1^ mg^−1^ of protein), the difference (3155 ± 561 nmol) corresponding to threonine-derived acetate, (ii) a 17.3% reduction of glucose contribution to acetate production (1240 ± 250 nmol *versus* 1499 ± 235 nmol of excreted molecules h^−1^ mg^−1^ of protein, *P*-value < 0.001) and (iii) a 1.6-fold and 2.3-fold increase of succinate and pyruvate excretion from glucose, probably as a consequence of the observed reduction of glucose-derived acetate production (Fig. 2B and Table 1). More importantly, these metabolite analyses show that, in the presence of equal amounts of threonine and glucose, the amino acid is the main source of acetate, which contributes approximately 2.5-fold more to acetate production than glucose (3155 ± 561 nmol *versus* 1240 ± 250 nmol of excreted molecules h^−1^ mg^−1^ of protein). Unfortunately, we could not determine the rate of acetate production from threonine in the absence of glucose, because procyclic cells die within two hours in PBS/threonine conditions. To get closer to physiological conditions, the wild type procyclics were incubated in the presence of low amounts of threonine (0.2, 0.5 and 1 mM) with equimolar amounts of glycerol, a carbon source probably present in the insect vector instead of glucose. In these conditions, threonine remains the main acetate source regardless of the quantities of carbon sources available (Fig. S1).

**Table 1:** Excreted end-products of glucose and threonine metabolism by procyclic *T. brucei* cell lines

Cell line^a^,^b^	*n*^c^	Excreted molecules from [^13^C-U]-glucose^d^				
		Acetate	Succinate	Pyruvate	Alanine	TOTAL
		nmol h^−1^ mg^−1^ of protein (% of excreted molecules)				
WT^e^	20	1499 ± 235 (72.0)	433 ± 151 (20.8)	150 ± 97 (7.2)	ND^f^	2082 ± 382 (100)
*^RNAi^*TDH.ni	3	1358 ± 65 (65.5)	418 ± 30 (20.1)	300 ± 19 (14.4)	ND	2076 ± 93 (100)
*^RNAi^*TDH.i	3	1505 ± 235 (67.4)	447 ± 64 (20.0)	281 ± 101 (12.6)	ND	2233 ± 413 (100)
*^RNAi^*PDH.ni	3	1132 ± 247 (62.0)	408 ± 66 (22.3)	286 ± 63 (15.7)	ND	1826 ± 369 (100)
*^RNAi^*PDH.i	3	71 ± 12 (3.4)	508 ± 94 (24.4)	1373 ± 269 (66.0)	131 ± 43 (6.3)	2083 ± 381 (100)
*^RNAi^*TDH/*^RNAi^*PDH.ni	3	992 ± 92 (61.5)	415 ± 32 (25.7)	206 ± 81 (12.8)	ND	1613 ± 167 (100)
*^RNAi^*TDH/*^RNAi^*PDH.i	3	119 ± 34 (8.7)	404 ± 45 (29.6)	780 ± 40 (57.2)	62 ± 61 (4.5)	1366 ± 124 (100)
Δ*ach*/*^RNAi^*ASCT.ni	5	1051 ± 150 (50.6)	617 ± 186 (29.7)	408 ± 160 (19.6)	ND	2076 ± 364 (100)
Δ*ach*/*^RNAi^*ASCT.i (4d)	4	200 ± 97 (13.9)	564 ± 237 (39.2)	674 ± 98 (46.9)	ND	1438 ± 283 (100)
Δ*ach*/*^RNAi^*ASCT.i (9d)	2	123 (7.6)	592 (36.6)	726 (44.8)	179 (11.1)	1621 (100)

Cell line^a^,^f^	*n*^c^	Excreted acetate from threonine^h^	Excreted molecules from [^13^C-U]-glucose^d^					Ratio acetate (Thr)/(Glu)^i^
			Acetate	Succinate	Pyruvate	Alanine	TOTAL	
		nmol h^−1^ mg^−1^ of protein (% of excreted molecules)							
WT^e^	38	3155 ± 561	1240 ± 250 (53.1)	695 ± 156 (29.8)	346 ± 155 (14.8)	53 ± 64 (2.3)	2335 ± 368 (100)	2.54
*^RNAi^*TDH.ni	3	881 ± 62	1356 ± 149 (64.4)	430 ± 121 (20.5)	318 ± 58 (15.1)	ND^f^	2104 ± 229 (100)	0.65
*^RNAi^*TDH.i	3	ND	1463 ± 136 (64.1)	490 ± 16 (21.5)	329 ± 88 (14.4)	ND	2282 ± 237 (100)	<0.001
Δ*pdh*	6	3688 ± 273	ND	678 ± 232 (47.3)	621 ± 64 (43.4)	132 ± 45 (9.3)	1431 ± 120 (100)	>100
*^RNAi^*PDH.ni	9	3201 ± 322	1162 ± 94 (52.0)	608 ± 33 (27.2)	402 ± 42 (18.0)	61 ± 31 (2.8)	2233 ± 177 (100)	2.75
*^RNAi^*PDH.i	3	3134 ± 369	83 ± 30 (3.9)	847 ± 126 (39.7)	1058 ± 165 (49.6)	144 ± 81 (6.8)	2132 ± 278 (100)	37.8
Δ*pdh*/*^RNAi^*TDH.ni	3	1850 ± 70	ND	778 ± 75 (34.9)	1309 ± 79 (58.7)	144 ± 46 (6.4)	2231 ± 139 (100)	>100
Δ*pdh*/*^RNAi^*TDH.i	3	57 ± 37	ND	458 ± 88 (21.0)	1580 ± 289 (72.3)	149 ± 127 (6.8)	2187 ± 468 (100)	>100
*^RNAi^*TDH/*^RNAi^*PDH.ni	3	710 ± 66	951 ± 100 (52.5)	505 ± 31 (27.9)	330 ± 35 (18.2)	25 ± 43 (1.4)	1811 ± 118 (100)	0.75
*^RNAi^*TDH/*^RNAi^*PDH.i	3	64 ± 19	142 ± 34 (6.4)	545 ± 68 (24.4)	1488 ± 140 (66.7)	56 ± 32 (2.5)	2230 ± 223 (100)	0.45
Δ*ach*/*^RNAi^*ASCT.ni	6	1199 ± 236	492 ± 96 (20.4)	797 ± 229 (33.0)	1042 ± 289 (43.2)	83 ± 113 (3.4)	2415 ± 369 (100)	2.44
Δ*ach*/*^RNAi^*ASCT.i (4d)	4	103 ± 66	119 ± 92 (6.9)	511 ± 196 (29.5)	1004 ± 238 (58.0)	97 ± 131 (5.6)	1732 ± 199 (100)	0.87
Δ*ach*/*^RNAi^*ASCT.i (9d)	2	57	34 (1.8)	665 (34.8)	1006 (52.6)	208 (10.9)	1914 (100)	1.67
Δ*pepck*	11	1714 ± 215	1030 ± 136 (81.3)	52 ± 40 (4.1)	105 ± 86 (8.3)	80 ± 97 (6.3)	1267 ± 158 (100)	1.66
Δ*tdh*/TDH	3	1361 ± 46	1109 ± 92 (50.1)	782 ± 32 (35.4)	320 ± 26 (14.5)	ND	2211 ± 97 (100)	1.23
Δ*asct*	9	3304 ± 365	1070 ± 163 (53.2)	624 ± 180 (31.0)	320 ± 84 (15.9)	ND	2014 ± 242 (100)	3.09

a i: RNAi cell lines tetracycline-induced during 5 to 10 days depending on the cell line and the experiments; .ni: non-induced RNAi cell lines.

b Cells incubated in PBS containing 4 mM [U-^13^C]-glucose.

c Number of experiments.

d Mean ± SD of 3 to 38 experiments (nmol h^−1^ mg^−1^ of protein) and the percent of each excreted molecules (values into brackets) are presented.

e EATRO1125.T7T cell line.

f Non-detectable.

g Cells incubated in PBS containing 4 mM [U-^13^C]-glucose and 4 mM threonine.

h Mean ± SD of 3 to 38 experiments (nmol h^−1^ mg^−1^ of protein) are presented.

i Ratio between glucose-derived and threonine-derived acetate excreted.

The extracellular PBS medium of trypanosome cell lines incubated in the presence of 4 mM [U-^13^C]-glucose, in the presence or not of 4 mM threonine, was analysed by ^1^H-NMR spectrometry to detect and quantify excreted end-products.

### Threonine is the main source for lipid biosynthesis

**Figure 3:**
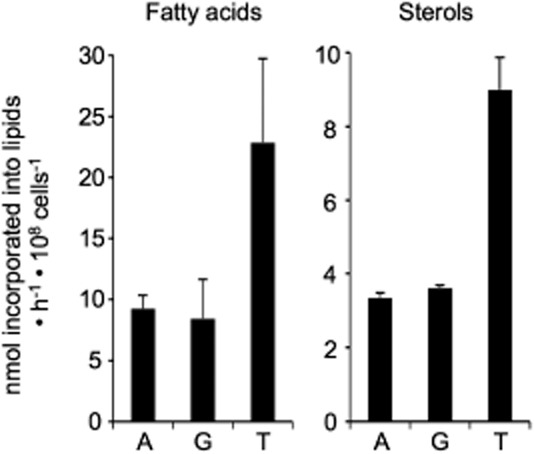
[1-^14^C]-acetate, d-[U-^14^C]-glucose and l-[U-^14^C]-threonine incorporation into lipids of the procyclic trypanosomes. ^14^C-labelled fatty acid methyl esters and sterols were separated by HPTLC after transesterification and analysed as described in the Experimental procedures section. The EATRO1125.T7T procyclic cells were incubated 16 h in SDM79 containing 4 mM glucose and 4 mM threonine with 1 mM acetate and [1-^14^C]-acetate (lanes A), d-[U-^14^C]-glucose (lanes G) or l-[U-^14^C]-threonine (lanes T) prior to lipid extraction. The data are expressed as nmol of acetate, glucose or threonine (radioactive and non-radioactive molecules) incorporated into fatty acids or sterols in 10^8^ cells per hour. Error bars indicate mean ± SD of 5 independent experiments.

We previously reported that acetate produced in the mitochondrion of procyclic trypanosomes is essential for *de novo* fatty acid biosynthesis (Riviere *et al*., 2009). To compare the relative contribution of threonine and glucose to lipid biosynthesis, we determined the radiolabel incorporation from d-[U-^14^C]-glucose, [U-^14^C]-threonine or [1-^14^C]-acetate into fatty acids and sterols in wild-type procyclic cells incubated for 16 h in SDM79 medium containing equal amounts of glucose and threonine (4 mM). Fatty acid methyl esters (FAMEs) from total lipids were formed by trans-esterification, separated from sterols by HPTLC, and the radiolabelling of FAMEs and sterols was then determined (Fig. 3). The amounts of threonine incorporated into fatty acids and sterols is ∼ 2.5-fold higher than the amount of glucose incorporated into these molecules, indicating that threonine is the preferred carbon source for lipid biosynthesis. This is in agreement with the observed ∼ 2.5 times higher contribution of threonine to the production of acetate (Table 1), which is an essential precursor of *de novo* fatty acid biosynthesis (Riviere *et al*., 2009).

### Threonine degradation is a non-essential mitochondrial pathway in standard growth conditions

**Figure 4:**
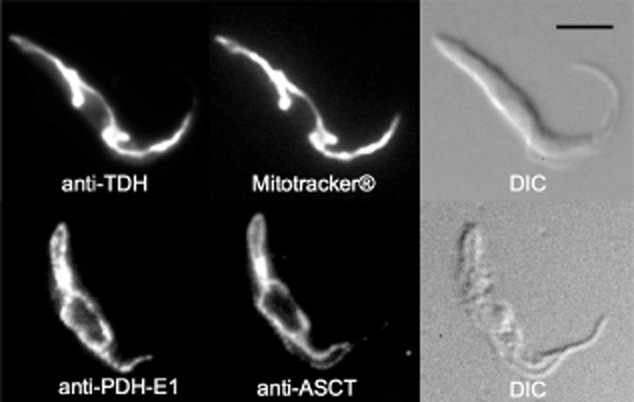
Immunolocalization of TDH and PDH. Procyclic cells were stained with rabbit anti-TDH (Alexa 488 channel) and MitoTracker® (top panels) or mouse anti-PDH-E1α (Alexa 488 channel) and rabbit anti-ASCT (Alexa 594 channel) (lower panels). Differential interference contrast (DIC) of cells is shown to the right of each panel. Scale bar, 5 μm.

According to the current model, glucose-derived acetyl-CoA is produced from pyruvate by the action of the PDH complex (step 3 in Fig. 1). Acetyl-CoA is then converted in the mitochondrion into acetate by ASCT (step 4) and ACH (step 5) (Millerioux *et al*., 2012). A pathway to produce acetate from threonine has not been experimentally validated to date but the *T. brucei* genome contains the coding capacity to degrade threonine to acetyl-CoA by threonine 3-dehydrogenase (TDH, EC 1.1.1.103; step 1) and 2-amino-3-ketobutyrate coenzyme A ligase (EC 2.3.1.29, step 2). Antibodies raised against the *T. brucei* TDH protein expressed in *Escherichia coli* recognize a single 36.5 kDa protein in Western blots, corresponding to the protein's calculated molecular weight (36.96 kDa). Immunofluorescence analyses revealed colocalization with the mitochondrion-specific dye Mitotracker® Red CMXRos (Invitrogen) (Fig. 4, upper panel) and known mitochondrial proteins including the E1α subunit of PDH and ASCT (Fig. 4, lower panel). The mitochondrial localization of TDH is consistent with a 24 amino acid N-terminal mitochondrial targeting signal predicted by MitoProt (http://ihg.gsf.de/ihg/mitoprot.html) with a high probability (0.82).

**Figure 5:**
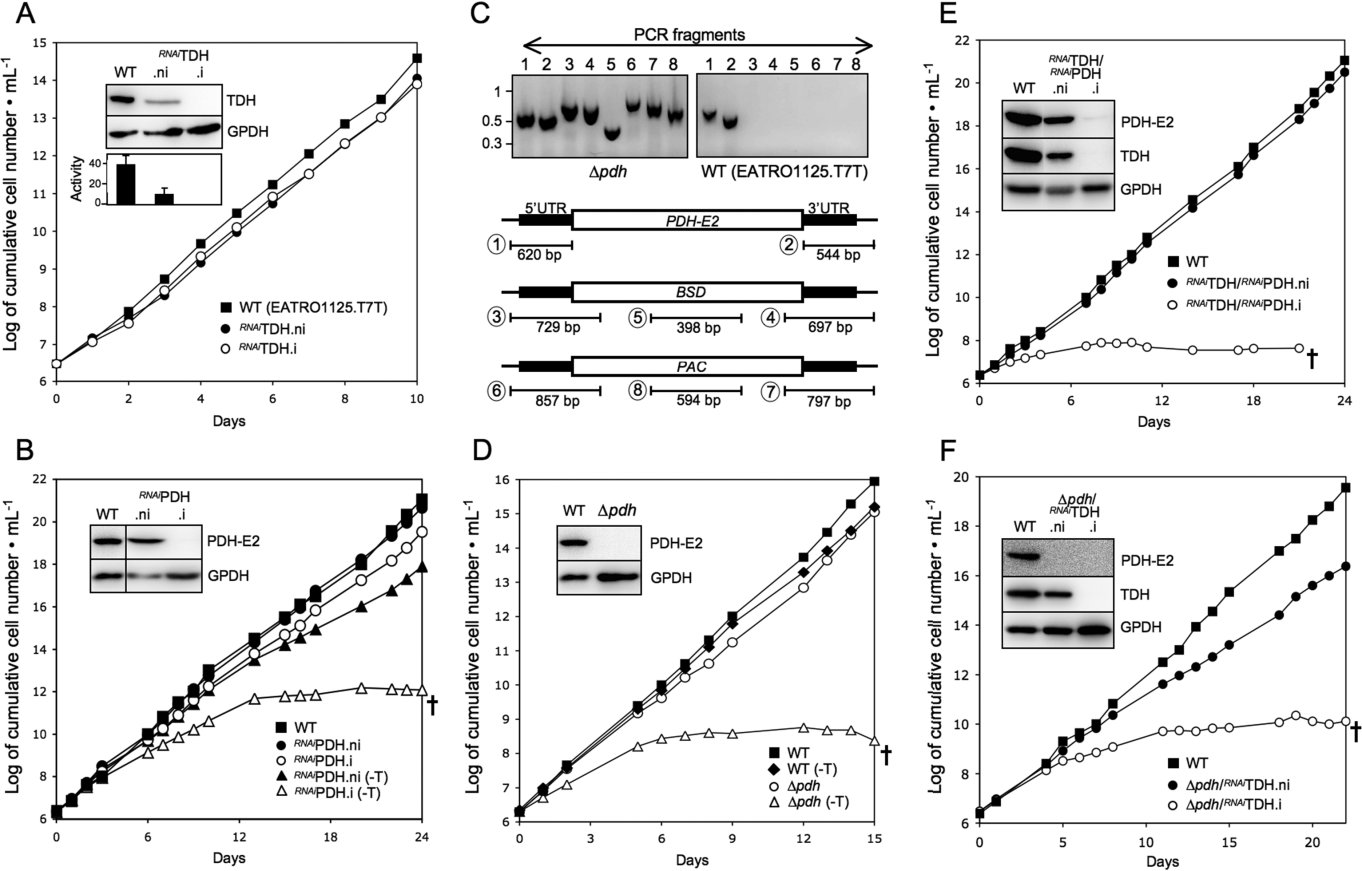
Analysis of mutant cell lines. The figure shows growth curve of the Δ*pdh* null mutant (D) and of the ^*RNAi*^TDH (A), ^*RNAi*^PDH (B), ^*RNAi*^TDH/^*RNAi*^PDH (E) and Δ*pdh*/^*RNAi*^TDH (F) mutant cell lines incubated in the presence (.i, ○) or in the absence (.ni, •) of tetracycline, and compared with the parental EATRO1125.T7T cell line (WT, ▪). Cells were maintained in the exponential growth phase (between 10^6^ and 10^7^ cells ml^−1^) and cumulative cell numbers reflect normalization for dilution during cultivation. All cell lines were grown in SDM79 medium; the ^*RNAi*^PDH (B), Δ*pdh* (D) and WT (D) cell lines were also grown in threonine-depleted conditions (-T). Crosses indicate that all cells were dead. The insets in panels A–B and D–F show Western blot analyses of the parental (WT) and mutant cell lines with the immune sera indicated in the right margin. The lower inset in panel A shows the TDH activity (milliunits mg^−1^ of protein) normalized with the malic enzyme activity measured in the same samples. Panel C, shows a PCR analysis of genomic DNA isolated from the parental EATRO1125.T7T (WT) and Δ*pdh* cell lines. Amplifications were performed with primers based on sequences that flank the 5′UTR and 3′UTR fragments used to target the *PDH-E2* gene depletion (black boxes) and internal sequences from the *PDH-E2* gene (PCR products 1 and 2), the blasticidin resistance gene (*BSD*, PCR products 3–5) or the puromycin resistance gene (*PAC*, PCR products 6–8). As expected, PCR amplification using primers derived from the *PDH-E2* gene and drug resistant genes were only observed for the parental EATRO1125.T7T and Δ*pdh* cell lines respectively.

Downregulation of the *TDH* gene in the EATRO1125.T7T background (*^RNAi^*TDH cell line) showed no growth phenotype upon tetracycline induction (Fig. 5A), although the TDH enzyme activity and the TDH protein are no longer detectable up to 12 days after tetracycline induction (Fig. 5A, insets). Metabolite profiling of the tetracycline-induced *^RNAi^*TDH.i cell line (.ni and .i stand for non-induced and tetracycline-induced respectively) incubated in the presence of 4 mM of d-[U-^13^C]-glucose and unenriched threonine showed a complete abolition of threonine-derived acetate production, while the rate of acetate production from glucose reached the value observed for the wild-type cells incubated in the absence of threonine (1463 nmol ± 136 *versus* 1499 ± 235 nmol of excreted molecules h^−1^ mg^−1^ of protein) (Fig. 2C and Table 1). This demonstrates that TDH is the only route through which threonine is metabolized to acetate. The increase of glucose-derived acetate production in the non-induced *^RNAi^*TDH.ni cell line is probably a consequence of the 2.9-fold decrease in threonine-derived acetate production (Table 1), that correlates with a 3.9-fold reduction of TDH activity (Fig. 5A, inset), presumed to arise from leaky RNAi expression even under non-induced conditions. The data demonstrate that TDH and the threonine degradation pathway are not essential for growth of the parasite, nor is growth of WT cells affected by removing threonine from the medium (see Fig. 5D).

### Inhibition of both threonine and glucose degradation is synthetically lethal

To completely abolish acetate production from both glucose and threonine metabolism, two complementary approaches were taken. First, expression of the E2 subunit of PDH (PDH-E2) was successfully downregulated in the *^RNAi^*PDH cell line (Fig. 5B, inset) and both *PDH-E2* alleles were replaced by the BSD and PAC markers in the Δ*pdh* cell line (Fig. 5C and D). Neither cell line is viable in threonine-depleted medium, while their growth is not affected in standard threonine-rich medium (Fig. 5B and D). Second, RNAi-mediated downregulation of TDH was performed in the *^RNAi^*PDH and Δ*pdh* backgrounds. Both the Δ*pdh*/*^RNAi^*TDH and *^RNAi^*TDH/*^RNAi^*PDH cell lines die upon tetracycline induction in standard rich medium (Fig. 5E and F), confirming that abolition of acetyl-CoA/acetate production from glucose and threonine is lethal for the procyclic trypanosomes grown in glucose-rich conditions. Addition of 5 mM acetate in the medium does not rescue growth of the *^RNAi^*TDH/*^RNAi^*PDH mutant (data not shown), suggesting that acetate or acetyl-CoA have to be produced in the mitochondrion to feed essential pathway(s), such as biosynthesis of mitochondrial fatty acids by the prokaryotic-like type II fatty acid synthase (Guler *et al*., 2008). In addition, acetate may participate in the generation of the mitochondrial membrane potential if exported from the mitochondrion in its protonated form (Bringaud *et al*., 2010).

The NMR metabolite profiling of the PDH mutant cell lines (*^RNAi^*PDH.i and Δ*pdh*) incubated in the presence of both of d-[U-^13^C]-glucose and unenriched threonine, revealed that glucose-derived acetate production is 14-fold reduced (*^RNAi^*PDH.i) compared with the parental cells, or not detectable (Δ*pdh*). As expected, threonine-derived acetate production is not affected in these mutants (Table 1, Fig. 2D). In agreement with the current model of glucose and threonine metabolism, acetate production from both carbon sources is almost completely abolished in the Δ*pdh*/*^RNAi^*TDH.i and*^RNAi^*TDH/*^RNAi^*PDH.i cell lines (Table 1, Fig. 2E). The rate of pyruvate production is considerably increased in all of these PDH-depleted mutants (up to 4.6-fold), while an increase in succinate production is moderate (up to 1.2-fold). As expected, pyruvate, the substrate of the targeted enzyme, accumulates in the PDH-depleted cell lines and is excreted. Succinate production occurs further upstream in the pathway (see Fig. 1) and is not significantly affected by PDH-depletion.

### Glucose- and threonine-derived acetate branches share the same last step

We recently demonstrated that glucose-derived acetate is produced from acetyl-CoA by the action of two redundant enzymes, acetate:succinate CoA-transferase (ASCT) and acetyl-CoA thioesterase (ACH) (Millerioux *et al*., 2012). RNAi downregulation of *ASCT* expression in the ACH null background (Δ*ach*/*^RNAi^*ASCT.i cell line) is lethal and abolished acetate production from glucose. NMR metabolite profiling of the Δ*ach*/*^RNAi^*ASCT.i cell line incubated in the presence of 4 mM of d-[U-^13^C]-glucose and unenriched threonine, showed 36.5- and 55.4-fold reduction of acetate production from each of these carbon sources, respectively, after 9 days of tetracycline induction (Fig. 2F and Table 1). The ∼ 2.5-fold reduction of acetate production from glucose (492 ± 96 nmol *versus* 1240 ± 250 nmol of excreted molecules h^−1^ mg^−1^ of protein) and threonine (1199 ± 236 nmol *versus* 3155 ± 561 nmol of excreted molecules h^−1^ mg^−1^ of protein) in the uninduced Δ*ach*/*^RNAi^*ASCT cell line is due to RNAi leakage in addition to *ACH* gene deletion (Millerioux *et al*., 2012). As expected, reduction of glucose-derived acetate production induced a notable increase in pyruvate excretion (threefold), even before tetracycline induction. These data clearly demonstrate, for the first time, that the last step of acetate production catalysed by ASCT and ACH is shared by the glucose and threonine degradation pathways.

### Glycine production from threonine

Linstead *et al*. previously showed that threonine is converted into equimolar amounts of acetate and glycine (Linstead *et al*., 1977). By ^1^H-NMR, a single glycine resonance (3.52 ppm) is detected near one of the two threonine doublets (3.56 and 1.28 ppm) (see Fig. S2A, inset). Although the close proximity of these resonances (3.52 and 3.56 ppm) affects the quantitative analyses of glycine production and a 2D ^1^H-NMR approach does not separate the glycine and threonine resonances (data not shown), this semi-quantitative analysis reveals that the ratio between threonine-derived glycine and acetate is 0.88 ± 0.14, confirming that both end-products are secreted in similar amounts (see Fig. S2A). This ratio is 10-fold increased in the Δ*ach*/*^RNAi^*ASCT.i cell line (9.51 ± 3.30), while it remains the same in the *^RNAi^*TDH.i and *^RNAi^*TDH/*^RNAi^*PDH.i mutants (see Fig. S2B and C; quantitative data not shown). The higher production of glycine compared with acetate in the Δ*ach*/*^RNAi^*ASCT.i, is in agreement with the current model (Fig. 1), since glycine is produced before conversion of acetyl-CoA into acetate by ASCT and ACH. In addition, we observed that a significant amount of threonine-derived glycine is incorporated into trypanothione, which results from the condensation of spermidine with two glutathione molecules, the latter being a glycine containing tripeptide. LC-MS metabolic profiling of intracellular metabolites of the wild-type procyclic cells incubated with [^15^N]-threonine show that 26% of the trypanothione molecules are ^15^N-enriched (data not shown) and given that cysteine, glutamate and spermidine, the other components of trypanothione are not labelled beyond the natural abundance of ^15^N, this label must correspond to the incorporated glycine.

### TDH expression is downregulated in the Δ*pepck* mutant

**Figure 6:**
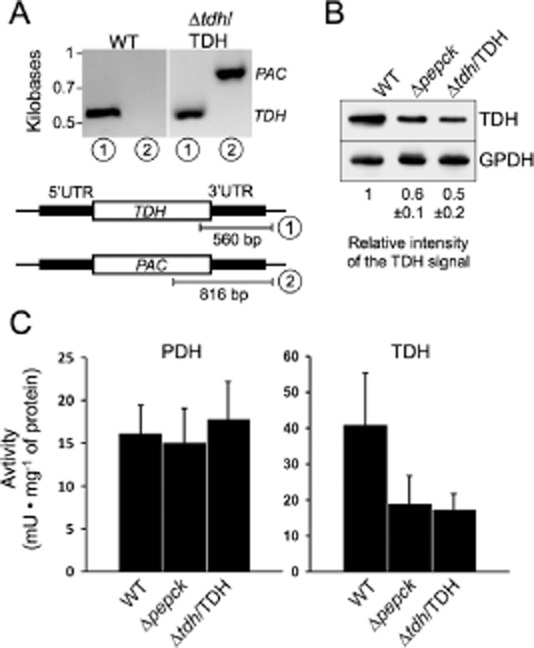
TDH expression and activity are reduced in the Δ*pepck* cell line. Demonstration of the single *TDH* allele replacement in the Δ*tdh*/TDH cell line is presented in panel A. PCR analysis of genomic DNA isolated from the WT and Δ*tdh*/TDH cell lines was performed with primers based on sequences that flank the 3′UTR fragment used to target depletion of one *TDH* allele (black boxes) and internal sequences from the *TDH* gene (PCR product 1) or the *PAC* (PCR product 2). As expected, PCR amplification using the primer derived from the *PAC* gene was only observed for the Δ*tdh*/TDH cell line. In panel B, expression of TDH and glycerol 3-phosphate dehydrogenase (GPDH) was analysed by Western blotting with specific immune sera. Ratio between the TDH and GPDH signals, indicated below the blot, represents a mean ± SD of 3 different experimental duplicates, with an arbitrary value of 1 for the parental cells (WT). Panel C shows the PDH (right panel) and TDH (left panel) activities (milliunits mg^−1^ of protein), normalized with the malic enzyme activity measured in the same samples, i.e. WT, Δ*pepck* and Δ*tdh*/TDH cell lines.

**Figure 7:**
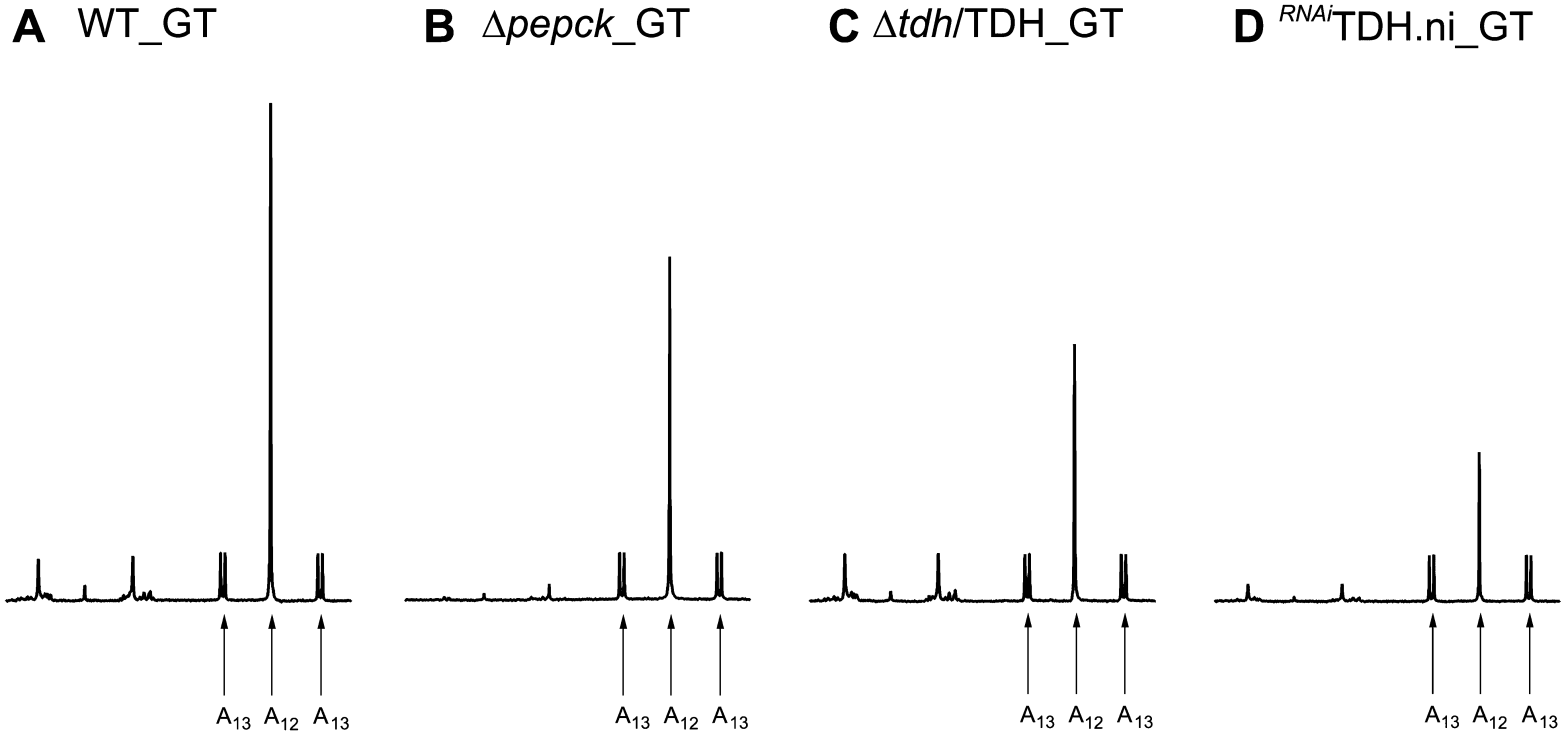
Threonine-derived acetate excretion is reduced in the Δ*pepck* cell line. Metabolic end-products (succinate, pyruvate, and acetate) excreted by the procyclic wild-type (A), Δ*pepck* (B), Δ*tdh*/TDH (C) and ^*RNAi*^TDH.ni (D) cell lines from d-[U-^13^C]-glucose and threonine was determined by ^1^H-NMR. The cells were incubated in PBS containing 4 mM d-[U-^13^C]-glucose and 4 mM threonine. Each spectrum corresponds to one representative experiment from a set of at least 3. A part of each spectrum ranging from 1.6 ppm to 2.6 ppm is shown. For the resonances assignment, see Fig. 2.

In the procyclic trypanosomes, glucose breakdown produces PEP, which is converted into two major excreted end-products, i.e. acetate and succinate (Fig. 1). We previously manipulated its intermediate metabolism by redirecting the glycolytic flux towards acetate production (Ebikeme *et al*., 2010). This was performed by deleting the *PEPCK* gene (Δ*pepck* cell line), which encodes the first enzymatic step of both succinate branches (step 8). To identify possible protein expression adaptations in response to these metabolic changes, we compared the proteome of the Δ*pepck* and wild-type cells by 2D-DiGE, as previously described (Alban *et al*., 2003). This analysis highlighted a protein species, which was 1.5-fold (*P* = 4.0 e^−6^) downregulated in the Δ*pepck* cell line (Fig. S3). This protein was confidently identified, by tandem mass spectrometry and Mascot searching, as TDH (MOWSE score 485, 56% peptide coverage). Modulation of TDH was confirmed by the 1.7-fold reduction of TDH in the Δ*pepck* cell line observed by Western blotting with the anti-TDH and anti-GPDH (control) immune sera (Fig. 6B). The TDH activity is also downregulated in the Δ*pepck* cell line by a similar order of magnitude (2.1-fold) (Fig. 6C). Interestingly, downregulation of TDH expression and activity induces a reduction of threonine's contribution to acetate production. Indeed, the ratio between threonine-derived and glucose-derived acetate production is 1.5-fold reduced in the Δ*pepck* cell line compared with the wild-type cells (1.66 *versus* 2.54) (Fig. 7A and B and Table 1). This correlation is consistent with the analysis of the *^RNAi^*TDH.ni cell line showing 3.9-fold and 3.6-fold reduction of TDH activity and threonine-derived acetate production respectively (Figs 5A and 7D, Table 1). In addition, ^15^N-incorporation into trypanothione is approximately twofold reduced in the Δ*pepck* cell line compared with the wild-type parasites (data not shown).

**Figure 8:**
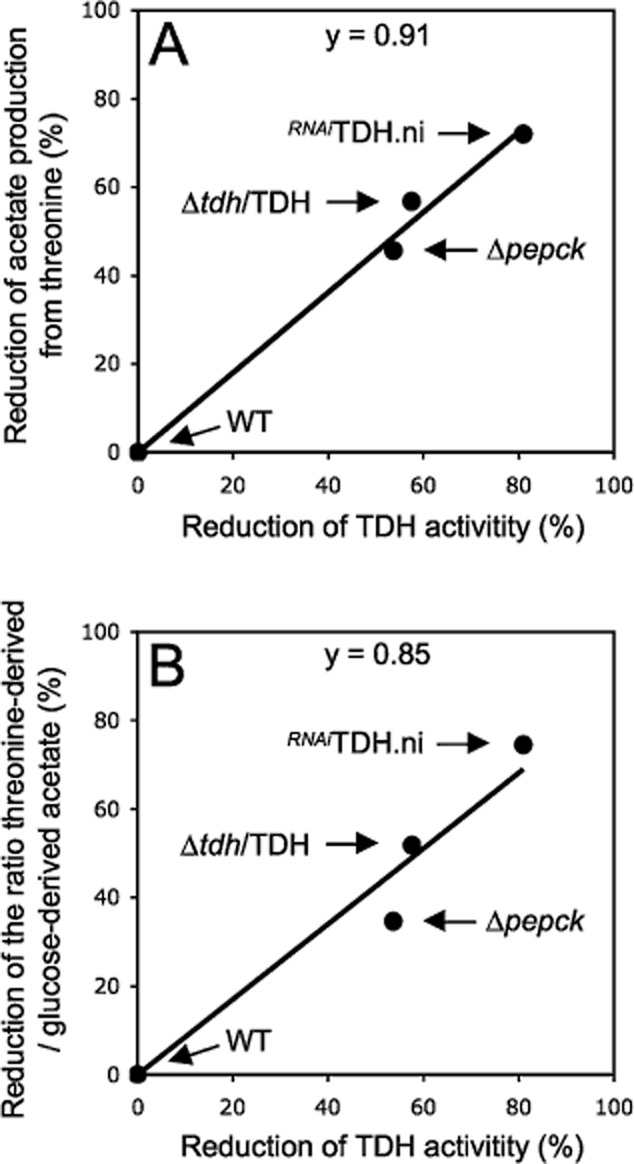
Correlation between TDH activity and acetate production. These graphs show, the percentage of reduction of acetate production from threonine (panel A) and the percentage of reduction of the ratio between threonine-derived acetate and glucose-derived acetate (panel B), as a function of the TDH activity, in the Δ*pepck*, Δ*tdh*/TDH and ^*RNAi*^TDH.ni mutants compared with the wild-type cells. The slope of the curves (y) is close to 1, suggesting a direct correlation between TDH activity and acetate production from threonine.

To further confirm the direct correlation between the TDH protein/activity and threonine-derived acetate production, we generated a Δ*tdh*/TDH cell line in which one allele of the *TDH* gene was replaced by the puromycin marker (Fig. 6A). The Δ*tdh*/TDH cell line showed a twofold and 2.3-fold reduction of the TDH protein levels and activity respectively (Fig. 6B and C), associated to a twofold reduction of threonine's contribution to overall acetate production (Fig. 7C and Table 1), as observed for the Δ*pepck* cell line. Figure 8A illustrates the direct correlation of TDH activity and threonine-derived acetate production in the wild-type, Δ*pepck*, Δ*tdh*/TDH and *^RNAi^*TDH.ni cell lines. Collectively these data demonstrate that TDH is a rate-limiting step of the threonine degradation pathway, which is downregulated in the Δ*pepck* cell line to reduce contribution of threonine in acetate production.

**Figure 9:**
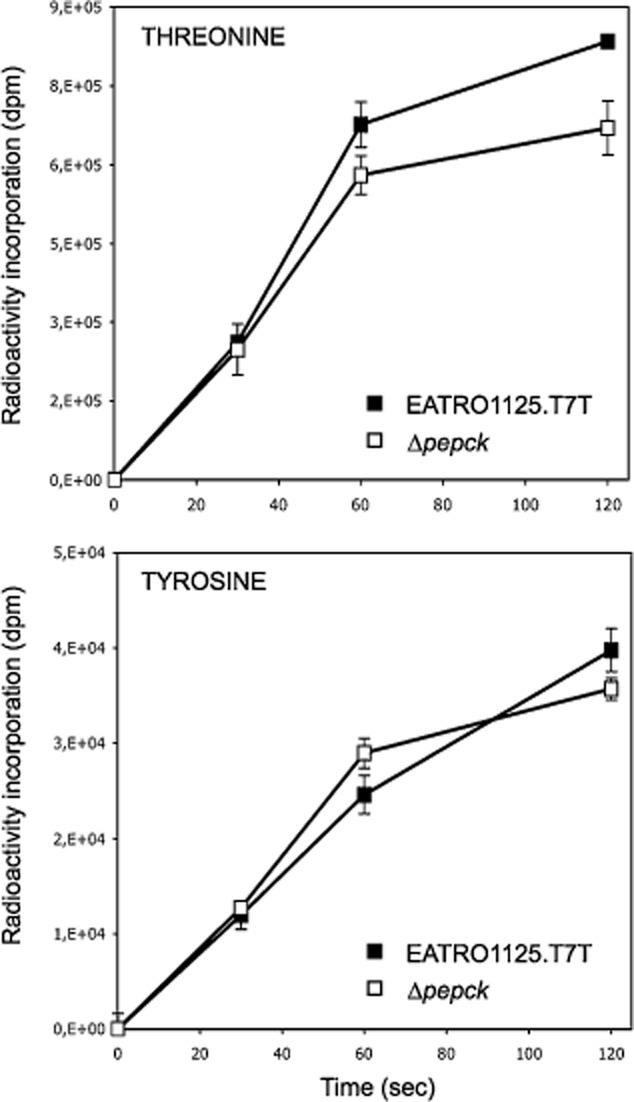
Threonine uptake. Incorporation of threonine and tyrosine in the EATRO1125.T7T (black squares) and Δ*pepck* (white square) cell lines over a two min time period.

Uptake of threonine was investigated in the wild-type and Δ*pepck* cell lines and found that threonine uptake is similar in both cell lines, as was tyrosine uptake (Fig. 9), indicating that transport activity is not affected by *PEPCK* gene deletion.

## Discussion

Threonine is a major carbon source consumed by procyclic trypanosomes and is converted into equimolar amounts of glycine and acetate (Cross *et al*., 1975; Linstead *et al*., 1977; and this present analysis). In contrast to glucose and proline, this amino acid alone cannot sustain growth of the parasite because it is not a good source for ATP production (Lamour *et al*., 2005). However, it is the amino acid by far the most rapidly consumed by procyclic trypanosomes grown in standard SDM79 medium, with a consumption rate similar to glucose (Lamour *et al*., 2005). Here we show that threonine is the preferred carbon source for lipid biosynthesis, as previously proposed (Klein and Linstead, 1976), contributing to both the fatty acid and sterol biosynthetic pathways ∼ 2.5 times more than glucose contributes to the same pathways. This implies that it is degraded to acetyl-CoA and acetate to feed the initial steps of sterol and fatty acid biosynthesis, which are respectively located in the mitochondrion (Carrero-Lerida *et al*., 2009; Mazet *et al*., 2011) and the cytosol (Vigueira and Paul, 2011) (see Fig. 1). We previously showed that glucose-derived acetate is produced exclusively inside the mitochondrion from acetyl-CoA by ASCT and ACH (Millerioux *et al*., 2012). Here we show that the same two enzymes produce acetate from threonine, suggesting that the mitochondrion is the site for all acetyl-CoA and acetate production in procyclic form *T. brucei*. Thus, acetyl-CoA production and initiation of sterol biosynthesis take place in the same subcellular compartment; the first three steps of sterol biosynthesis from acetyl-CoA being catalysed by mitochondrial enzymes, i.e. thiolase, 3-hydroxy-3-methyl-glutary-CoA (HMG-CoA) synthetase and HMG-CoA reductase (Carrero-Lerida *et al*., 2009; Mazet *et al*., 2011). However, acetyl-CoA must be transferred from the mitochondrion to the cytosol to feed the first step of the fatty acid biosynthetic pathways catalysed by the cytosolic acetyl-CoA carboxylase (Vigueira and Paul, 2011). This is achieved by the so-called acetate shuttle, a trypanosomatid specific pathway involving the cytosolic AceCS (Riviere *et al*., 2009) (see Fig. 1).

Procyclic trypanosomes are not dependent on threonine for optimal growth in the standard glucose rich-medium, where glucose is sufficient to feed lipid biosynthesis. However, the role of threonine in lipid biosynthesis may be essential in the insect vector (*Glossina* species), which becomes a glucose-free environment 15–30 min after injection of the blood meal. The doubling time of procyclic cells grown in medium depleted of glucose, threonine and pyruvate is significantly reduced compared with glucose/pyruvate-depleted conditions (18.5 h *versus* 11.6 h), but not abolished (Y. Millerioux, M. Mazet, P. Morand and F. Bringaud, unpubl. data). It is important to note that our threonine-depleted medium still contains approximately 15 μM threonine derived from the 10% supplement of fetal calf serum [mammalian blood contains ∼ 150 μM threonine (Cynober, 2002; Ohnishi *et al*., 2012)]. This suggests that either 15 μM threonine is sufficient to feed *de novo* lipid biosynthesis or additional carbon source(s) can be used for lipid biosynthesis, such as other ketogenic amino acids present in the standard medium as well as in the insect vector (Balogun *et al*., 1969; Balogun, 1974), as long as the corresponding degradation pathway is functional in the parasite.

*Leishmania* genomes do not contain the threonine degradation pathways (orthologues of *TDH* and 2-amino-3-ketobutyrate coenzyme A genes are absent), suggesting that they have developed other acetyl-CoA/acetate production pathway(s). *Leishmania mexicana* promastigotes produce acetate from aspartate (Saunders *et al*., 2011), although this pathway has not been described in procyclic trypanosomes (Berriman *et al*., 2005). *Leishmania* promastigotes also produce acetyl-CoA from β-oxidation in their glycosomes (Gannavaram *et al*., 2012; Colasante *et al*., 2013), although this pathway has also not been demonstrated in trypanosomes so far (Colasante *et al*., 2006). In *Leishmania*, it has also been shown that leucine is the main precursor for sterol biosynthesis using an acetyl-CoA independent pathway. The same pathway has been shown to be only weakly active in *T. cruzi* but has not been investigated in *T. brucei* so far (Ginger *et al*., 1999; 2000[Bibr b27],[Bibr b28]). Trypanosomes and *Leishmania* have clearly developed different strategies to provide acetyl-CoA, which is a key metabolite linking many biosynthetic and catabolic pathways.

In wild-type procyclic trypanosomes, a direct correlation is observed between the ratio of the specific activity of TDH and PDH (2.53; 41.0 mU mg^−1^ of protein *versus* 16.2 mU mg^−1^ of protein) and between the ratio of the threonine-derived and glucose-derived acetate productions (2.54; 3155 nmol *versus* 1240 nmol of excreted molecules h^−1^ mg^−1^ of protein). This suggests that the metabolic flux through the glucose and threonine branches is controlled primarily by PDH and TDH activities. The analysis of the *Δtdh*/TDH cell line, lacking one *TDH* allele, strongly supports this hypothesis since a 58% reduction of the TDH activity leads to a 57% reduction of acetate production from threonine. The same correlation is observed in the *^RNAi^*TDH.ni cell line (Fig. 8). A consequence of this metabolic control may be a fine-tuning of acetate production from the main acetate source, i.e. threonine, in response to the cellular acetyl-CoA or acetate demand and/or accumulation. Interestingly, TDH downregulation was observed in the Δ*pepck* cell line, which showed a redistribution of the glucose metabolic flux towards acetate excretion, as a consequence of abolition of succinate production from glucose (see Fig. 1) (Ebikeme *et al*., 2010). As observed for the Δ*tdh*/TDH cell line, the 52% reduction of the TDH activity in the Δ*pepck* mutant is correlated with the 46% reduction of threonine-derived acetate production. We propose that redirection of the glycolytic metabolic flux towards acetate production in the Δ*pepck* cells induces a signal to downregulate TDH expression, in order to reduce the contribution of threonine to acetyl-CoA and acetate production. Collectively, these data indicate that metabolic flux through the glucose-derived acetate branch downregulates TDH activity, thus reducing the contribution of the threonine degradation pathway in acetyl-CoA and acetate production.

We have previously demonstrated how the availability of glucose causes diminished activity of proline dehydrogenase and proline transport (Lamour *et al*., 2005). Here it seems that TDH (but not threonine transport) is downregulated when glycolytic flux is redirected towards acetate production. The mechanistic basis of this apparent cross-talk between metabolic pathways has not yet been investigated.

In mammalian cells, TDH is a key enzyme of acetyl-CoA metabolism under tight metabolic control. In the rat liver, for example, regulation of TDH is essential for fatty acid metabolism and the enzyme is a target of feedback inhibition by various compounds derived from its major product, acetyl-CoA (Guerranti *et al*., 2001), with the ketone body β-hydroxybutyrate being the most efficient. Ketone bodies (β-hydroxybutyrate, acetoacetate and acetone) are water-soluble compounds that are produced as by-products when fatty acids are broken down for energy in the liver. β-hydroxybutyrate and acetoacetate are used as energy sources in the heart and brain while acetone is a waste product excreted from the body. The activity of the *T. brucei* TDH is not affected by up to 10 mM β-hydroxybutyrate, nor acetate, β-hydroxybutyryl-CoA or acetyl-CoA (data not shown), suggesting a mechanism of regulatory control that differs from that in rat liver. Post-transcriptional and post-translational regulation of TDH has recently been described in the mouse embryonic stem cells to modulate somatic cell reprogramming (Han *et al*., 2013). In *T. brucei* reduced TDH activity is correlated with a reduction of TDH protein amounts in the Δ*pepck* cell line, which suggests a post-transcriptional, translational or post-translational regulation of its gene expression. It will be of significant interest to determine the mechanisms underpinning this regulated gene expression. Future metabolomic, proteomic and transcriptomic analyses will be useful in determining mechanisms that underlie reduced expression of TDH in the Δ*pepck* cell line, or reduced proline dehydrogenase and proline uptake in wild-type *T. brucei* procyclics grown in glucose-depleted conditions. It has recently been demonstrated that decarboxylated S-adenosylmethionine (dcAdoMet) contributes directly to regulation of its own cellular abundance in trypanosomes. When levels of this metabolite decrease, a corresponding increase in translation of the RNA encoding the regulatory protein called prozyme occurs and this in turn activates S-adenosylmethionine decarboxylase, the enzyme responsible for production of dcAdoMet (Xiao *et al*., 2013) thus restoring levels of the diminished metabolite.

Threonine degradation to acetate has also been described previously in bloodstream forms of *T. brucei* (Linstead *et al*., 1977). To date, the relevance of this pathway to parasite viability and infectivity have not been addressed and equivalent experiments to those described here, on mammalian infective forms of *T. brucei* are presently in progress to explore acetate production in bloodstream form organisms.

## Experimental procedures

### Growth and maintenance of trypanosomes

The procyclic form of *T. brucei* EATRO1125.T7T (*TetR-HYG T7RNAPOL-NEO*) was cultured at 27°C in SDM79 medium containing 10% (v/v) heat-inactivated fetal calf serum and 3.5 mg ml^−1^ hemin (Brun and Schonenberger, 1979) or in a threonine-depleted SDM79, which still contains threonine (∼ 15 μM) but only that coming from heat-inactivated fetal calf serum.

### Gene knockout

Replacement of the *PEPCK* gene (Tb927.2.4210, http://www.genedb.org/genedb/tryp/) by the blasticidin (BSD) and puromycin (PAC) resistance markers via homologous recombination was described before (Δ*pepck* cell line) (Ebikeme *et al*., 2010). Replacement of the E2 subunit gene of the PDH complex (*PDH-E2*: Tb927.10.7570) by BSD and PAC resistance markers via homologous recombination was performed with DNA fragments containing a resistance marker gene flanked by the PDH-E2 UTR sequences. Briefly, the pGEMt plasmid was used to clone an HpaI DNA fragment containing the BSD and PAC resistance marker gene preceded by the PDH-E2 5′-UTR fragment (565 bp) and followed by the PDH-E2 3′-UTR fragment (464 bp). One allele of the *TDH* gene (Tb927.6.2790) was replaced by the PAC resistance marker *via* homologous recombination with a DNA fragment containing a resistance marker gene flanked by the TDH 5′-UTR (621 bp) and 3′-UTR (494 bp) sequences. The *PDH-E2* knockout and replacement of one *TDH* allele were generated in the EATRO1125.T7T parental cell line, which constitutively expresses the T7 RNA polymerase gene and the tetracycline repressor under the control of a T7 RNA polymerase promoter for tetracycline inducible expression (*TetR-HYG T7RNAPOL-NEO*) (Bringaud *et al*., 2000). Transfection and selection of drug-resistant clones were performed as reported previously (Bringaud *et al*., 1998). Transfected cells were selected in SDM79 medium containing hygromycin B (25 μg ml^−1^), neomycin (10 μg ml^−1^), blasticidin (10 μg ml^−1^) and/or puromycin (1 μg ml^−1^). The selected cell lines *TetR-HYG T7RNAPOL-NEO Δpdh-e2::BSD/Δpdh-e2::PAC* and *TetR-HYG T7RNAPOL-NEO Δtdh::PAC/TDH* are called Δ*pdh* and *Δtdh/TDH* respectively.

### Inhibition of gene expression by RNAi

Inhibition of gene expression by RNAi in procyclic forms (Ngo *et al*., 1998) was performed by expression of stem-loop ‘sense/anti-sense’ RNA molecules of the targeted sequences (Bringaud *et al*., 2000) introduced in the pLew100 expression vector (kindly provided by E. Wirtz and G. Cross) (Wirtz *et al*., 1975), as previously described. To downregulate expression of the *TDH* gene, the pLew-TDH-SAS plasmid was constructed to target a 408 bp fragment of the *TDH* gene (from position 398 bp to 806 bp). Briefly, a PCR-amplified 501 bp fragment, containing the antisense TDH sequence with restriction sites added to the primers was inserted into the HindIII and BamHI restriction sites of the pLew100 plasmid. Then a PCR-amplified fragment containing the sense TDH sequence (429 bp) was inserted upstream of the anti-sense sequence, using HindIII and XhoI restriction sites (XhoI was introduced at the 3′-extremity of the antisense PCR fragment). The resulting plasmid (pLew-TDH-SAS) contains a sense and antisense version of the targeted gene fragment, separated by a 64 bp fragment, under the control of the PARP promoter linked to a prokaryotic tetracycline (Tet) operator. For downregulation of the *PDH-E2* gene expression by RNAi, the ‘sense/anti-sense’ cassette of the pLew-PDH-E2-SAS plasmid described before (Coustou *et al*., 2008), was introduced into pHD1336 vector, which contains the blasticidin resistance gene, to generate the pHD-PDH-E2-SAS plasmid. The *^RNAi^*TDH and Δ*pdh*/*^RNAi^*TDH cell lines were produced by introducing the pLew-TDH-SAS plasmid in the EATRO1125.T7T and Δ*pdh* cell lines respectively. The *^RNAi^*TDH/*^RNAi^*PDH double mutant was produced by transfecting the *^RNAi^*TDH cell line with the pHD-PDH-E2-SAS plasmid. Transfected cells were selected in SDM79 medium containing, phleomycin (5 μg ml^−1^; *^RNAi^*TDH), blasticidin (10 μg ml^−1^; *^RNAi^*PDH), phleomycin and blasticidin (*^RNAi^*TDH/*^RNAi^*PDH), or puromycin (1 μg ml^−1^), blasticidin and phleomycin (Δ*pdh*/*^RNAi^*TDH), in addition to hygromycin B (25 μg ml^−1^) and neomycin (10 μg ml^−1^). Downregulation by RNAi of the *ASCT* (Tb11.02.0290) gene expression in the ACH (Tb927.3.4260) null background (Δ*ach*/*^RNAi^*ASCT cell line) is described elsewhere (Millerioux *et al*., 2012). Induction of double-stranded RNA expression was performed by addition of 1 μg ml^−1^ tetracycline.

### Enzyme assays

TDH enzyme activity was adapted from Linstead *et al*. (1977) using a spectrophotometric assay. Briefly, cells were washed in PBS and resuspended in hypotonic lysis buffer (5 mM Na_2_HPO_4_, 0.3 mM KH_2_PO_4_) and sonicated (5 sec at 4°C). The enzyme assay contained 0.5 mM NAD^+^, 200 mM Tris HCl pH 8 and 30 mM threonine. As a control, sonicated crude extracts of trypanosomes were tested for malic enzyme (EC 1.1.1.40) and PDH activities (Klein *et al*., 1975; Else *et al*., 1994).

### Production of TDH and PDH antibodies

A recombinant fragment containing the full-length *TDH* gene or a fragment of the E1α subunit gene of the PDH complex (PDH-E1α: Tb927.3.1790), corresponding to positions 415 to 1138 bp of the PDH-E1α gene, were inserted into the NdeI and BamHI restriction sites of the pET28a (TDH) and pET16b (PDH-E1α) expression vectors (Novagen), to express in the BL21 *E. coli* cell the TDH and PDH-E1α proteins preceded by a N-terminal histidine tag (6 histidine codons). Cells were harvested by centrifugation, and recombinant proteins purified by nickel chelation chromatography (Novagen) from the insoluble fraction according to the manufacturer's instructions. The anti-TDH immune serum was raised in rabbits by five injections at 15 day intervals of 100 μg of TDH-His recombinant nickel-purified proteins, emulsified with complete (first injection) or incomplete Freund's adjuvant (Proteogenix S.A.). For PDH-E1α antibodies, mice were immunized with 10 μg of recombinant nickel-purified proteins by injection, with complete (first injection) or incomplete Freund's adjuvant, into the peritoneum.

### Western blot analyses

Total protein extracts of wild-type or mutant procyclic form of *T. brucei* (5 × 10^6^ cells) were size-fractionated by SDS-PAGE (10%) and immunoblotted on Immobilon-P filters (Millipore) (Harlow and Lane, 1988). Immunodetection was performed as described (Harlow and Lane, 1988; Sambrook *et al*., 1989) using as primary antibodies, the rabbit anti-TDH (diluted 1:500), the rabbit anti-ASCT (diluted 1:100) (Riviere *et al*., 2004), the rabbit anti-GPDH (glycerol 3-phosphate dehydrogenase, EC 1.1.1.8; diluted 1:100) (Denise *et al*., 1999), the mouse anti-hsp60 (diluted 1:10 000) (Bringaud *et al*., 1995) and the mouse anti-PDH-E2 (diluted 1:500) (Ebikeme *et al*., 2010) and as secondary antibodies, anti-mouse or anti-rabbit IgG conjugated to horseradish peroxidase (Bio-Rad, 1:5000 dilution). Revelation was performed using the SuperSignal® West Pico Chemiluminescent Substrate as described by the manufacturer (Thermo Scientific). Alternatively, for quantitative analyses, revelation was performed using the Luminata™ Crescendo Western HRP Substrate (Millipore). Images were acquired and analysed with a KODAK Image Station 4000 MM and quantitative analyses were performed with the KODAK MI application.

### Immunofluorescence analyses

Log phase cells were fixed with formaldehyde as described before (Bringaud *et al*., 1998). Slides were incubated with rabbit anti-TDH (diluted 1:100) and Mitotracker®, or mouse anti-PDH-E1 (diluted 1:50) and rabbit anti ASCT (diluted 1:100) (Riviere *et al*., 2004) followed by ALEXA Fluor® 594-conjugated goat anti-mouse secondary antibody (diluted 1:100) and/or ALEXA Fluor® 488-conjugated goat anti-rabbit secondary antibody (diluted 1:100) (Molecular Probes), depending on the analysed cell. Cells were viewed with a Leica DM5500B microscope and images were captured by an ORCA®-R^2^ camera (Hamamatsu) and Leica MM AF Imaging System software (MetaMorph®) and merged in Adobe Photoshop on a Macintosh iMac computer.

### Proteomics analysis by 2D DiGE

Wild-type and Δ*pepck* cells were harvested from 4 independent cultures of each line and washed three times in cold phosphate buffered saline (PBS, pH 7.4). Difference gel electrophoresis analysis was performed as previously described (Daneshvar *et al*., 2012) and 2D gel spots were processed for protein identification as previously described (Bridges *et al*., 2008).

### NMR experiments

The 10^8^ *T. brucei* procyclic cells were collected by centrifugation at 1400 *g* for 10 min, washed once/twice with PBS and incubated for 6 h at 27°C in 5 ml of incubation buffer (PBS supplemented with 5 g l^−1^ NaHCO_3_, pH 7.4), with d-[U-^13^C]-glucose (4 mM) in the presence or the absence of threonine (4 mM). The integrity of the cells during the incubation was checked by microscopic observation. Fifty microlitres of maleate (20 mM) were added as internal reference to a 500 μl aliquot of the collected supernatant and ^1^H-NMR spectra were performed at 125.77 MHz on a Bruker DPX500 spectrometer equipped with a 5 mm broadband probe head. Measurements were recorded at 25°C with an ERETIC method. This method provides an electronically synthesized reference signal (Akoka *et al*., 1999). Acquisition conditions were as follows: 90° flip angle, 5000 Hz spectral width, 32 K memory size, and 9.3 s total recycle time. Measurements were performed with 256 scans for a total time close to 40 min. Before each experiment, phase of ERETIC peak was precisely adjusted. Resonances of obtained spectra were integrated and results were expressed relative to ERETIC peak integration. Protons linked to acetate carbon C2 generates by ^1^H-NMR five resonances, a single peak ([^12^C]-acetate) flanked by two doublets ([^13^C]-acetate). When d-[U-^13^C]-glucose is the only carbon source (no threonine), the central resonance (1.88 ppm) corresponding to [^12^C]-acetate, probably derived from an unknown internal carbon source. As a consequence it is not included in the glucose-derived acetate quantitative analyses (Fig. 2A). Similarly, the [^12^C]-succinate resonance (2.35 ppm, annotated S_12_) observed between both d-[U-^13^C]-glucose-derived [^13^C]-succinate resonances (2.23 ppm and 2.48 ppm, annotated S_13_) was not considered (Fig. 2A). The linear production of acetate and succinate throughout the experiment was confirmed by ^1^H-NMR quantification of the end-products excreted by the wild type trypanosomes incubated for 6 h in PBS containing 4 mM [U-^13^C]-glucose in the presence or absence of 4 mM threonine (Fig. S4). Data were analysed with the StatPlus package (AnalystSoft) of the Microsoft Excel software, using variance analysis followed by Student's *t*-test (unpaired, equal variances) to determine statistical differences in mean values as indicated in the text. Statistical differences are significant for *P*-values < 0.05.

### Lipid labelling from d-[^14^C]-glucose, l-[^14^C]-threonine and [^14^C]-acetate

The 10^8^ cells in the late exponential phase were incubated for 16 h in 5 ml of modified SDM79 medium containing 4 mM glucose and 4 mM threonine with 25 μCi d-[U-^14^C]-glucose (300 mCi mmol^−1^) or 25 μCi l-[U-^14^C]-threonine (175 mCi mmol^−1^). Alternatively, the cells were incubated in SDM79 medium containing 4 mM glucose, 4 mM threonine, 1 mM acetate and 10 μCi of [1-^14^C]-acetate (55.3 mCi mmol^−1^). Cells were checked microscopically for viability several times during the incubation. Subsequently, lipids were extracted by chloroform/methanol (2:1, v/v) for 30 min at room temperature, and then washed three times with 0.9% NaCl. The washed lipid extracts were then evaporated and lipids were dissolved in 1 ml of methanol/H_2_SO_4_ (40:1, v/v). The trans-esterification of the fatty acids of the lipids was done at 80°C for 60 min. After cooling the samples, 400 μl of hexane (99% pure) and 1.5 ml of H_2_O were added, and the mixture was homogeneized vigorously during 20 sec. The samples were then centrifuged 5 min at 1000 *g* to separate phases, and the hexane upper phases containing FAMEs and sterols were recovered without contact with the lower phases. FAMEs and sterols were loaded onto HPTLC plates developed in hexane/ethylether/acetic acid (90:15:2, v/v) and sterols (R_F_ 0.20) and FAMEs (R_F_ 0.90) were separated. They were identified by co-migration with known standards. Their radiolabelling was then determined with a STORM 860 (GE Healthcare).

### Mass spectrometry analysis of [^15^N]-threonine incorporation into intracellular metabolites

Procyclic trypanosomes were kept in log phase growth (below 2 × 10^7^ ml^−1^) in threonine-depleted SDM79 medium containing 5 mM [^15^N]-threonine (Cambridge Isotope Laboratories, 98% ^15^N-enriched). Intracellular metabolites were extracted by centrifugation as described before (Vincent *et al*., 2012). Samples were analysed on an Exactive Orbitrap mass spectrometer (Thermo Fisher) in both positive and negative modes (rapid switching), coupled to a U3000 RSLC HPLC (Dionex) with a ZIC-HILIC column (Sequant) as has previously been described (Vincent *et al*., 2010). All samples from an individual experiment were analysed in the same analytical batch and the quality of chromatography and signal reproducibility were checked by analysis of quality control samples, internal standards and total ion chromatograms. The few samples that displayed unacceptable analytical variation (retention time drift) were removed from further analysis. A standard mix containing approximately 200 metabolites (including members of the polyamine pathway) was run at the start of every analysis batch to aid metabolite identification. Data processing was performed as described before (Vincent *et al*., 2012).

### Transport assays

Parasites were harvested during the mid-log phase of growth, by centrifugation at 1250 rcf (2500 r.p.m.) for 10 min and washed three times with assay buffer (CBSS) by centrifugation at 1250 r.p.m. at 4°C. Parasites were resuspended in assay buffer at the density of 2 × 10^8^ cells ml^−1^ and kept on ice, and brought up to room temperature when the experiment was about to proceed. Uptake of radiolabelled compounds was determined using a derivation of the rapid oil/stop spin protocol, as previously described (Carter and Fairlamb, 1993). Transport was initiated with 100 μl of cells being mixed with 100 μl assay buffer, containing radiolabelled compounds (l-3-^3^H-threonine was from American Radiolabelled chemicals at 20 Ci mmol^−1^ and l-3,5-^3^H-tyrosine from Amersham Radiolabelled Chemicals at 49 Ci mmol^−1^). To separate trypanosome cells from radiolabelled amino acid solution, oil was used (1-Bromodo-decane, density: 1.066 g cm^−3^). The buffer was layered over a 90 μl cushion of oil. The oil's density, upon centrifugation, separates the trypanosome cells from the radioactive medium containing labelled amino acid. The tube was flash frozen in liquid nitrogen, and the pellet was separated from the remnants of the tube with a tube cutter, and lysed in 200 μl SDS (2%) and mixed with 3 ml scintillation fluid (Ecoscint A, National Diagnostics) and incorporated activity counted after 24 h (to avoid luminescence) using a liquid scintillation counter (Perkin-Elmer, liquid scintillation & luminescence counter, Microbeta). All experiments (at each time point for each substrate) were carried out, at least, in duplicate, and three times independently. Non-transported radiolabelled compounds, associated with cells or in interstitial spaces, were measured by performing control uptake determinations on ice (by commencing the reaction at a zero time point using cells, and buffer kept on ice to ensure, essentially, no uptake).
