# Supplementary material for: The threonine degradation pathway of the Trypanosoma brucei procyclic form: the main carbon source for lipid biosynthesis is under metabolic control
Source: Mol Microbiol. 2013 Aug 25;90(1):114–29. doi: 10.1111/mmi.12351 (PMC4034587; doi:10.1111/mmi.12351)
Supplement: Supplementary file 1 [file mmi0090-0114-sd1.pdf]

Figure S1

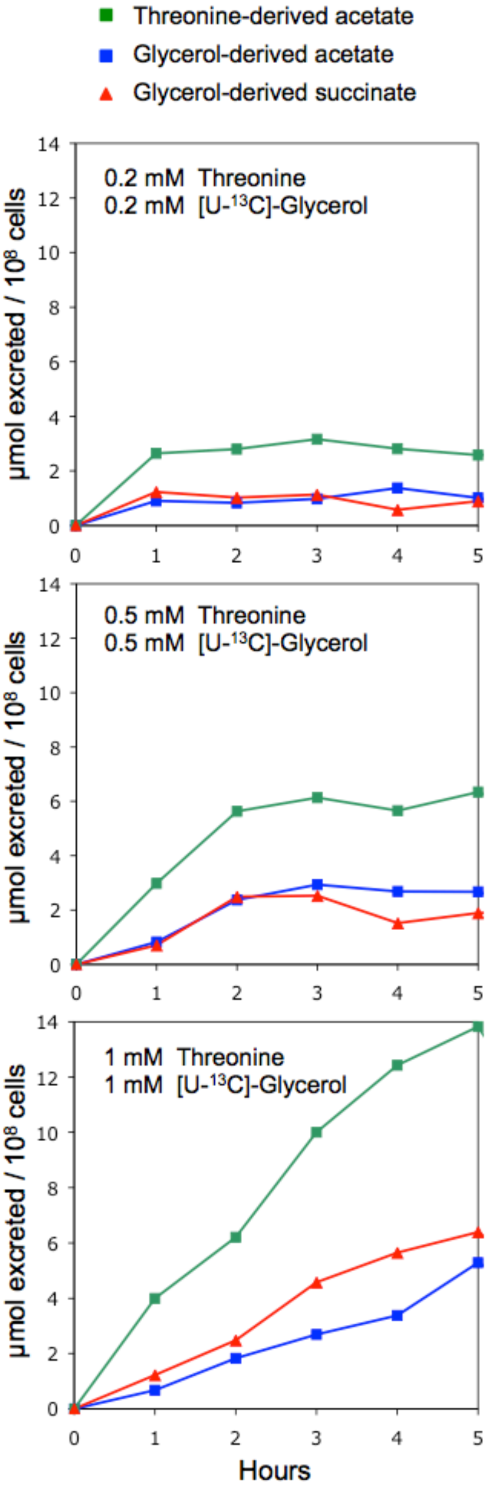

Kinetic of acetate and succinate production from low amounts of threonine and glycerol. The amounts of acetate and succinate produced by the wild type trypanosomes incubated in PBS containing 0.2, 0.5 or 1 mM of [U-^{13}C]-glycerol and threonine were determined by ^{1}H-NMR every hour during 5 h. The initial rate of acetate production for threonine is in the same range regardless of the amounts of carbon sources.

Figure S2

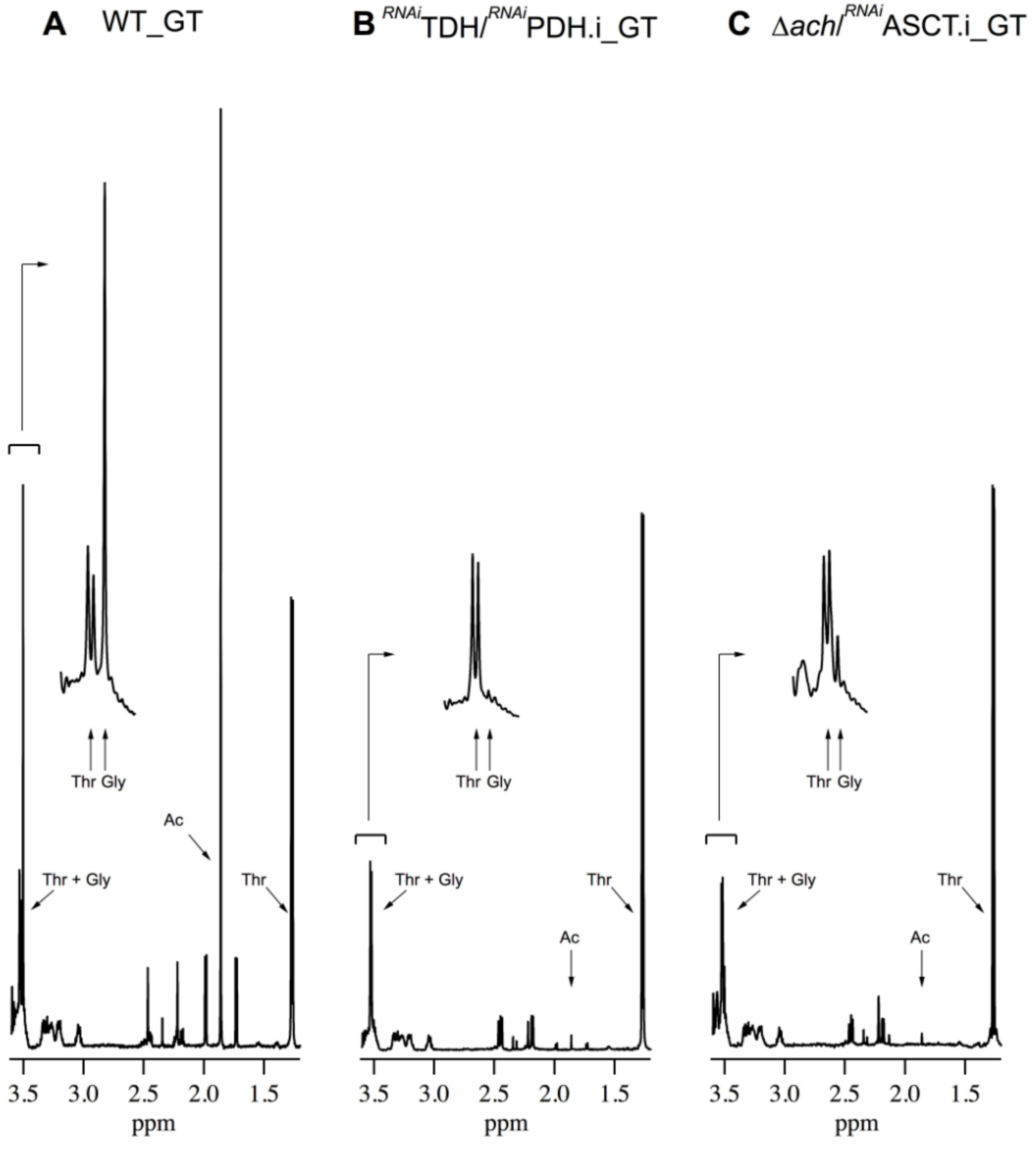

Production of glycine from threonine. Metabolic end-products (in particular acetate and glycine) excreted by the procyclic wild type cell line (A), the ^{RNAi}TDH/^{RNAi}PDH.i (B) and Δ*ach*/^{RNAi}ASCT.i (C) mutant from D-[U-^{13}C]-glucose and threonine was determined by ^{1}H-NMR. For more details see Fig. 2. A part of each spectrum ranging from 1.2 to 3.6 ppm is shown. A high resolution of the 3.5-3.6 ppm area is shown in the inset to separate the threonine doublet and the glycine singlet.

Figure S3

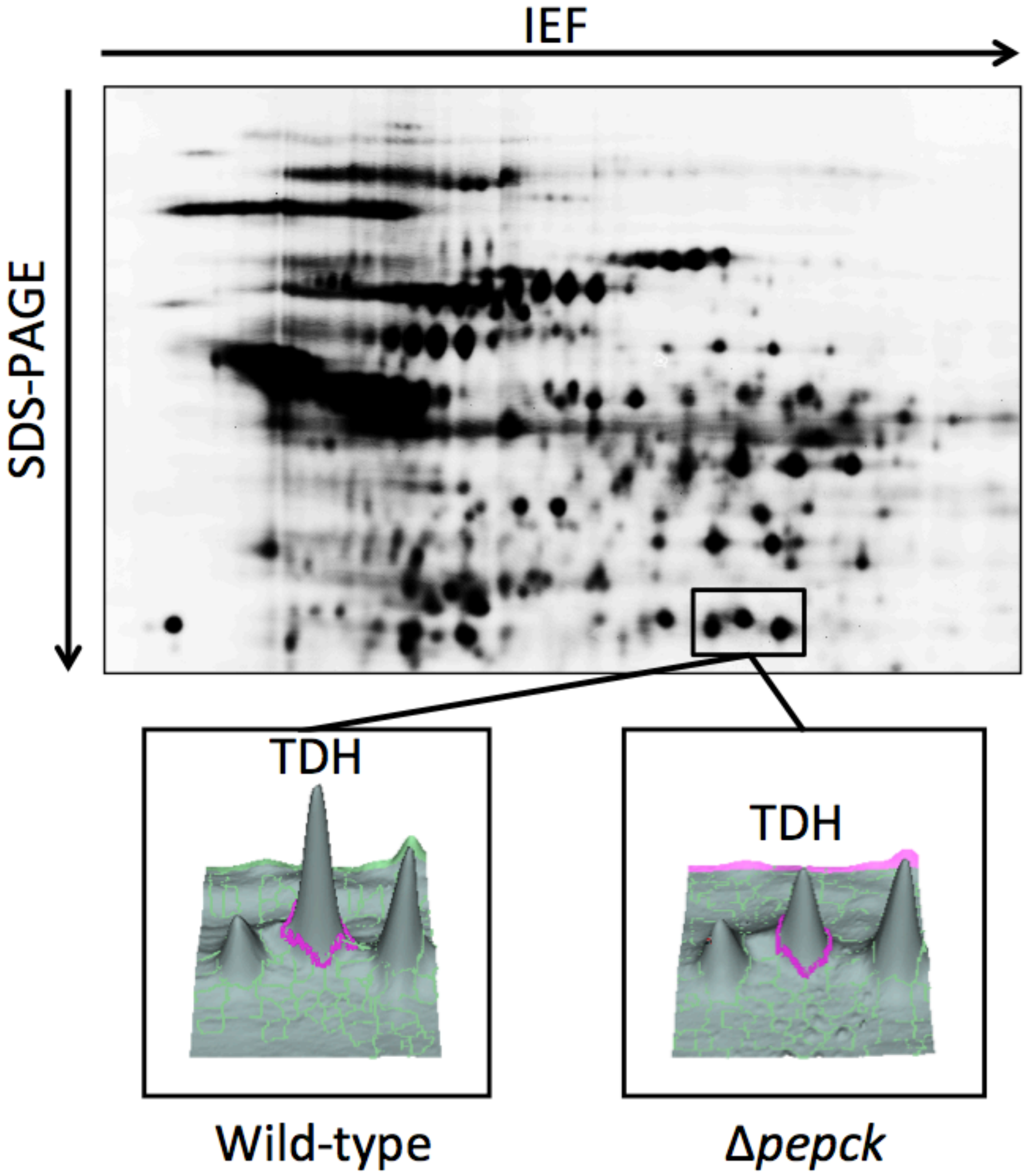

Difference gel electrophoresis (DIGE) analysis of wild type and Δ*pepck* procyclic form *T. brucei*. Four biological replicates of each line were compared by multiplex DIGE analysis, utilizing a pooled internal standard. A protein spot that was found to be down regulated in the Δ*pepck* by 1.5 fold (p=4.0 e-6; n=4) was identified as threonine dehydrogenase (TDH). Fold change indicates average change in standardized abundance. A representative 2D gel spot map is shown, with an inset box showing the location of the spot identified as TDH. The difference in spot intensity between wild type and Δ*pepck* is illustrated by 3 dimensional representations of spot volume.

Figure S4

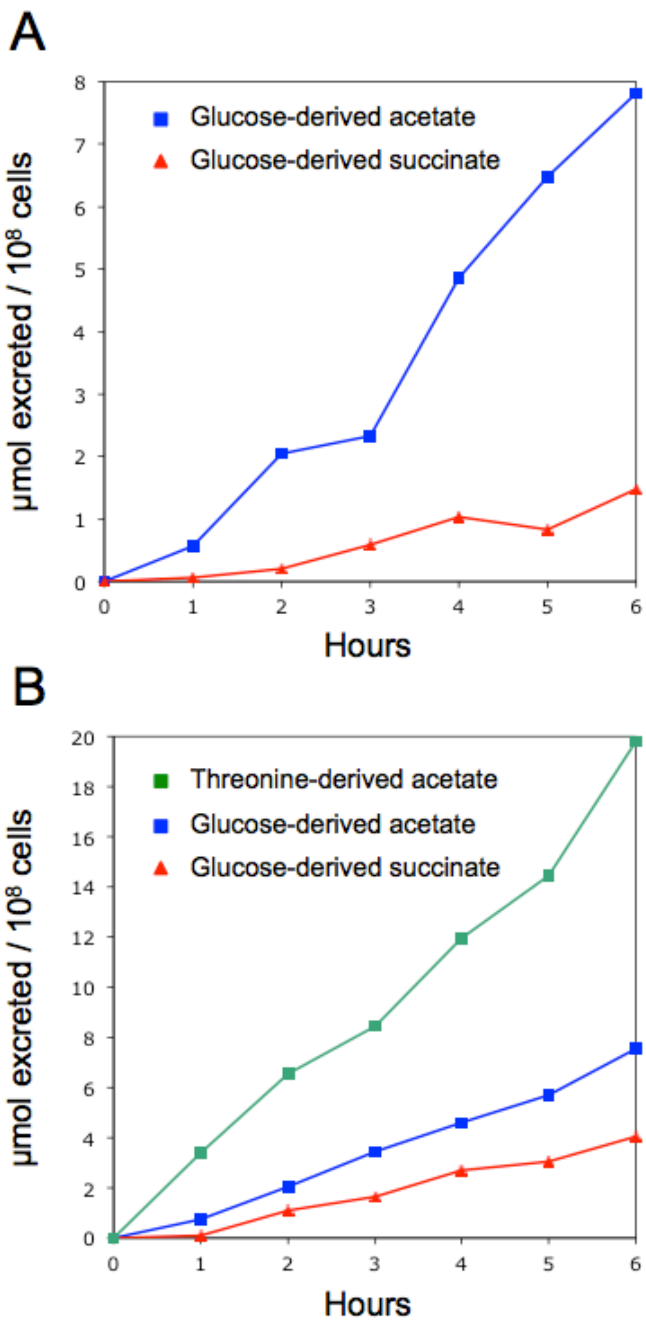

Kinetic of acetate and succinate production. The amounts of acetate and succinate produced by the wild type trypanosomes incubated in PBS containing [U-^{13}C]-glucose (A) or [U-^{13}C]-glucose and threonine (B) were determined by ^{1}H-NMR every hour during 6 h. Production of the excreted end product is linear during the whole experiment.
